# Anesthetic management of a pediatric patient with arginase-1 deficiency undergoing strabismus operation: a case report

**DOI:** 10.1186/s40981-019-0274-6

**Published:** 2019-08-29

**Authors:** Hideya Kato, Ken Kawaguchi, Teiji Sawa

**Affiliations:** Department of Anesthesiology, Kyoto Prefectural University of Medicine, 465 Kajiichō, Kawaramachi-Hirokoji, Kamigyō-ku, Kyōto-shi, Kyōto-fu 602-8566 Japan

**Keywords:** Arginase-1 deficiency, Hyperargininemia, Pediatric anesthesia

## Abstract

**Background:**

Urea cycle disorders are rare; arginase-1 deficiency is one of those extremely rare autosomal recessive metabolic disorders. Arginase-1 is one among the enzymes involved in the production of urea from ammonia in the liver, and its deficiency produces the characteristic feature, hyperargininemia.

**Case presentation:**

We report a case of a girl, aged 5 years and 10 months presenting with arginase-1 deficiency. The patient was scheduled to undergo strabismus surgery for intermittent exotropia under general anesthesia. Preoperative blood tests showed high serum arginine levels, but ammonia levels were within the normal range. Anesthesia was induced with sevoflurane and nitrous oxide via face mask and maintained with sevoflurane, fentanyl, and rocuronium. Vital signs were stable throughout the surgery. There was an intraoperative decrease in blood glucose levels (from 82 mg/dL to 42 mg/dL) that was treated with intravenous glucose. Arginine levels remained high after surgery; however, hyperammonemia did not develop. There were no complications and the patient was discharged on the following day.

**Conclusions:**

We successfully performed general anesthesia in a child with hyperargininemia. Only a few cases of arginase-1 deficiency had been reported and much remains unknown about its pathology. Therefore, information sharing among medical professionals is essential to customize the plan for the management of this disorder in patients.

## Background

The estimated prevalence of urea cycle disorders is 1 in 30,000 people. Of these, arginase-1 deficiency is reported in approximately 1 in 2.2 million people and is an extremely rare autosomal recessive metabolic disorder [1]. It is unknown how arginase-1 deficient patients would react to anesthesia. Here, we present a pediatric case of arginase-1 deficiency wherein general anesthesia was successfully performed.

## Case presentation

A girl aged 5 years and 10 months (weight, 13.9 kg, height, 100.5 cm) was scheduled to undergo strabismus surgery under general anesthesia. A diagnosis of arginase-1 deficiency was made based on high levels of serum arginine and ammonia, as well as on the results of genetic testing at the age of 4 years and 4 months. Her older sister was also diagnosed with the same disease. She has been treated with oral sodium phenylacetate and did not prefer to consume foods with animal protein/soy foods. Except short statue (− 2.4 S.D., standard deviation) and frequent ketotic hypoglycemic episodes, other symptoms were not observed. Serum arginine and ammonia levels were 811 nmol/ml (normal range, 71.8–230.4 nmol/mL) and 83 μg/dL (normal range, 19–79 μg/dL), respectively. Other laboratory data, chest X-ray, and electrocardiogram showed no abnormalities.

General anesthesia was induced with 50% nitrous oxide and with a stepwise increase in the concentration of sevoflurane administered via a face mask, followed by the insertion of intravenous line and tracheal intubation with rocuronium. Anesthesia was maintained with sevoflurane in 40% oxygen, fentanyl, and rocuronium. Acetate Ringer solution with 1% glucose was infused during anesthesia. Although circulatory and respiratory conditions were stable, blood glucose started to reduce from 82 mg/dL immediately after induction to 47 mg/dl 85 min after induction. It further decreased to 42 mg/dL despite the infusion of 5 mL 20% glucose. However, it subsequently increased to 127 mg/dL on administration of 5 mL 50% glucose. Surgery was completed without complications. The duration of surgery and anesthesia was 46 and 105 min, respectively. The tracheal tube was removed in the operating room. Blood glucose levels ranged between 90 and 100 mg/dL; serum arginine and ammonia levels were 623 and 73.8 nmol/ml, respectively, after surgery. Neither of them increased above the preoperative levels. The patient was discharged without complications the day after surgery. Patient’s consent was obtained for the publication of information using institutional written consent form.

## Discussion

**Fig. 1:**
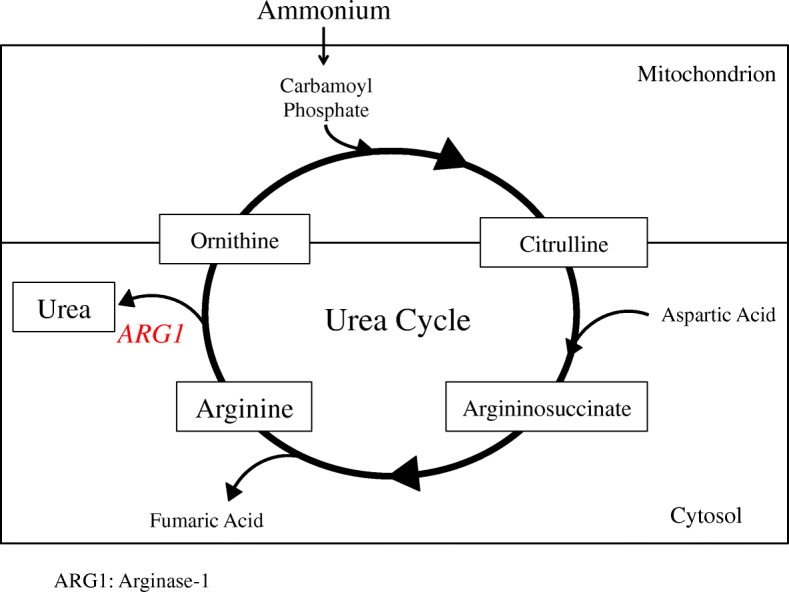
Urea cycle. Arginine levels in the blood are increased due to lack of arginase-1

The urea cycle is a pathway that produces urea from ammonia by transferring nitrogen, and consists of four amino acids: ornithine, citrulline, arginosuccinate, and arginine (Fig. 1). Any defect in the enzymes of this cycle leads to accumulation of the intermediates of the pathway. Arginase-1 deficiency causes hyperargininemia and hyperammonemia (Fig. 1). The differential diagnoses of childhood-onset hyperammonemia include congenital metabolic disorders, congenital vascular anomalies, severe infections, and drugs, such as anticonvulsants [2]. Any case with an increase in blood ammonia levels, a normal anion gap, and no hypoglycemia is suggestive of urea cycle disorders [3]. Genetic analysis would then be performed to confirm the diagnosis.

In the present case, although we performed a genetic analysis and made a definite diagnosis, ketotic hypoglycemic episodes, which are not typical of arginase-1 deficiency, were frequent. This is probably because the patient’s natural dietary intake was low; she appears to have unconsciously tried to avoid a high protein load. Typical clinical features included non-specific neurological symptoms like vomiting, poor suckling, tachypnea, convulsions, impaired consciousness, and behavioral and developmental disorders. The symptoms mentioned above are often attributable to hyperammonemia. In contrast, spastic paraplegia is found only in arginase-1 deficiency and is not seen in other urea cycle disorders. Spastic paraplegia is developed by elevated blood arginine levels that cause demyelination of upper motor neurons. In addition, arginine is a substrate for nitric oxide synthase and causes oxidative damage to neurons that may lead to spastic paraplegia [3]. Clinically, the disease is classified into the pre-symptomatic type (where the asymptomatic disease is detected during newborn screening tests), the newborn-onset type (where repeated episodes of vomiting, poor suckling, convulsions, and impaired consciousness occur in the neonatal period), and the late-onset type (where symptoms gradually appear after the neonatal period) [4].

Acute phase management uses intravenous administration of sodium benzoate (100–250 mg/kg/day) to excrete excess nitrogen [5]. If this does not decrease blood ammonia levels, blood purification techniques such as hemodialysis or hemodiafiltration are performed. In cases which are dialysis-dependent, living donor liver transplantation is indicated [6, 7]. Treatment for the chronic stage includes protein restriction in diet and amino acid therapy. As a drug therapy, oral sodium phenylbutyrate (200–300 mg/kg/day) is administered. Sodium phenylbutyrate rapidly turns into phenylbutyric acid in vivo and excretes glutamine, a precursor of ammonia, into urine by an alternative route different from the urea cycle. This has the effect of suppressing an increase in blood ammonia concentration [5].

Factors that can precipitate hyperammonemia in patients with arginase-1 deficiency include infections, fever, insufficient calorie intake, insufficient protein intake, general anesthesia, and high-stress conditions; all these conditions increase protein breakdown in the body [4]. Therefore, clinical management should center on preventing ammonia production due to enhanced protein catabolism.

To prevent hypoglycemia, preoperative planning should aim to minimize fasting time. For the same reason, infusion of extracellular fluid replacement solution containing glucose is recommended during surgery; we used an extracellular fluid replacement solution containing 1% glucose during surgery.

Arginine is a precursor of nitric oxide, which is well known to be a strong vasodilator. Therefore, patients with arginase-1 deficiency may develop significant hypotension during anesthetic induction [8]. To minimize any changes in circulation, we chose gradual induction with sevoflurane.

Dehydration enhances protein catabolism and worsens hyperammonemia; on the other hand, hyperammonemia may cause cerebral edema, which can be worsened by excessive infusion [8, 9]. Therefore, especially in cases where surgery is prolonged, highly invasive, or where severe blood loss is expected, it is necessary to monitor a dynamic index of preload such as pulse pressure variation or stroke volume variation.

Spastic paralysis is one of the symptoms of hyperargininemia [3]. The administration of muscle relaxants to patients with spastic paralysis is considered unsafe because of its unpredictable effects [8]. However, in the present case, we used a muscle relaxant and performed endotracheal intubation, as there was no history of spastic paralysis. This was also necessary to increase the safety of the procedure (especially since it was ophthalmic surgery).

Intraoperative monitoring of blood ammonia level is recommended and intravenous benzoic acid is used for emergency first-line management of hyperammonemia. Benzoic acid converts amino acids into hippuric acid, which can be directly excreted in the urine without following the natural pathway involving conversion to ammonia followed by metabolism in the urea cycle [8].

We had surmised that the patient’s history of frequent episodes of ketotic hypoglycemia was because of low dietary intake. However, the reason for the rapid drop in blood glucose levels following anesthetic induction is unclear.

## Conclusions

Arginase deficiency is an extremely rare disease with even fewer reports of anesthesia being performed in such patients. Nevertheless, we were able to safely perform general anesthesia in this case without any serious problems other than intraoperative hypoglycemia. Owing to its rarity, much remains unknown about the pathology of arginase deficiency. Therefore, information sharing between medical professionals is essential to establish norms to customize the disease management in these patients.

## Data Availability

A copy of the data presented in this report is available upon request.
